# Medication-related quality of life (MRQoL) in ambulatory older adults with multi-morbidity and polypharmacy

**DOI:** 10.1007/s41999-021-00573-6

**Published:** 2021-10-21

**Authors:** Emma L. M. Jennings, Denis O’Mahony, Paul F. Gallagher

**Affiliations:** 1 Department of Medicine (Geriatric Medicine), University College Cork, Wilton, T12 DC4A Cork Ireland; 2 Department of Geriatric Medicine, Cork University Hospital, Wilton, Cork Ireland; 3 Department of Medicine (Geriatric Medicine), Bon Secours Hospital, Cork, Ireland

**Keywords:** Multimorbidity, Polypharmacy, Quality of life, Older person

## Abstract

**Purpose:**

To assess medication-related quality-of-life (MRQoL) in multi-morbid older adults with polypharmacy and correlations with medications, frailty and health-related QoL.

**Methods:**

With a cross sectional study of multi-morbid geriatric medicine outpatients, we assessed MRQoL (MRQol-LSv1), frailty status, potentially inappropriate medications, Medication Adherence Rating Scale (MARS), health-related-QoL (Short-Form 12, SF12) and medication burden (Living with Medicines Questionnaire, LMQv2).

**Results:**

One-in-four (*n* = 59) of 234 outpatient attendees met inclusion criteria. Almost half (*n* = 106, 45%) were excluded due to cognition (MMSE < 26). Included participants (*n* = 27, mean age 80.2 years) experienced a median of 11 (IQR 9–13.5) co-morbidities and were prescribed a median of 10 (IQR 8–12.25) medications. Overall, MRQoL-LS.v.1 scores were low, suggesting good medication-related quality of life (median MRQoL-LS.v.1 score of 14, IQR 14–22). Correlations between MRQoL, number of daily medications, co-morbidity burden, LMQv2 score, SF12 scores and number of PIMs were non-significant.

**Conclusion:**

MRQoL-LSv.1 is unsuitable for most patients attending geriatric ambulatory services.

## Key summary points

**Aim:**

This study assesses medication-related quality-of-life using MRQoL-LS version 1 in ambulatory older adults with multi-morbidity and polypharmacy and explores potential correlations with medications, frailty and overall health-related QoL.

**Findings:**

Our sample of an ambulatory older patient cohort attending a specialist hospital-based Geriatric Medicine outpatient clinic experienced baseline polypharmacy, multi-morbidity and reported poor age adjusted health-related quality of life (HRQoL). However, there was no significant relationship between MRQoL-LS version 1 scores and number of chronic comorbid conditions, number of daily medications, number of potentially inappropriate medications taken daily or measured health-related QoL.

**Message:**

MRQoL-LS version 1 is not practical for most patients attending geriatric ambulatory services given the high proportion of patients attending with cognitive impairment. There is a need for a new medication-related QoL assessment tool that specifically addresses the impact of polypharmacy on QoL in multimorbid older people.

## Introduction

Although multi-morbidity and polypharmacy are commonplace in older people, medication-related outcomes affecting quality-of-life (QoL) are poorly characterised. Older people with multiple co-morbidities are frequently prescribed numerous medications. Previous research has highlighted the adverse consequences of potentially inappropriate prescribing including adverse drug events/reactions, avoidable iatrogenic morbidity, and greater healthcare resource utilisation [1]. However, patient reported outcome measures such as health-related quality of life (HRQoL) have received relatively little attention. QoL has a protective effect on mortality, which continues independent of age and sex [2]. Qualitative and quantitative measurement of QoL presents many challenges in older patients, particularly those with multimorbidity and cognitive impairment. Humanistic clinical outcomes, especially QoL, are rarely measured and poorly reported in published studies. Typically, QoL measures are reported as secondary endpoints and are usually underpowered, impeding definitive conclusions. Another consideration is the appropriateness of the selected QoL measurement tool in studies of pharmaceutical care (PC) interventions. HRQoL tools are commonly used to assess the impact of PC interventions despite their limited value as a sole humanistic measure; they also relate poorly to specific effects of medication burden on QoL [3].

Recently, a new medication-related QoL assessment tool called MRQoL-Life Scale version 1 (MRQoL-LSv1) has emerged. It employs a six-point Likert scale across 14 questions spanning three domains (role limitations due to medications, self-control and vitality) [4]. Lower MRQoL-LSv1 scores (range 14–84) represent better medication-related QoL. Although developed in a patient population mostly aged over 60 years with polypharmacy, the practicalities of its use as an outcome measure in older adult research are largely unknown. Krska et al. [5] have devised and validated another multidimensional tool (Living with Medicines Questionnaire (LMQ) version 2) to assess medication burden as an indicator of medication-related QoL, encompassing a five-point Likert scale across 60 questions spanning 8 domains, with higher scores indicating greater perceived medication burden (range 60–300). This tool is also largely untested in older adults.

Our aim was to assess the applicability of MRQoL-LSv1 as a patient-reported outcome in a sample of ambulatory older patients with polypharmacy and multi-morbidity. In addition, we examined statistical relationships between MRQoL-LSv1, medication adherence, medication burden, frailty, HRQoL and potentially inappropriate medications (PIMS).

## Methods

We undertook a cross sectional observational study of first-time ambulatory outpatient clinic attendees aged ≥ 65 years in a tertiary-teaching-hospital. Inclusion criteria were: polypharmacy (≥ 5 long-term daily medications), multi-morbidity (≥ 3 chronic conditions) and intact cognition (mini-mental state examination score ≥ 26/30 to facilitate consent and participant’s ability to accurately answer questionnaires). Demographic, medication and medical history data were recorded using a purpose-designed study pro-forma.

Frailty status was measured using the Edmonton Frail Scale (EFS; range 0–17) [6], higher scores indicate greater frailty; marked frailty was not an exclusion criterion. PIMs were defined by STOPP/START v.2 criteria [7]. We measured MRQoL using MRQoL-LS v.1 [4] and HRQoL in 12 domains using the Short-Form-12 (SF-12) questionnaire [8], higher scores indicating better perceived HRQoL. Also, negative age-specific mean-difference scores in SF-12 physical and mental health composite scale scores (SF12-PCS, SF12-MCS) indicate poorer health than those of similar age.

We measured medication burden using LMQ version 2 [5] addressing eight domains: patient–doctor relationship, interferences with daily life, practicalities, effectiveness, patient–pharmacist communication, acceptance of medicine use, autonomy/control over medicine use and concerns about potential harm; higher scores indicate higher medication burden. Drug adherence was measured using the Medication Adherence Rating Scale (MARS) [9]. Statements describing attitudes towards medication in the previous week were scored from 0 to 10, higher scores indicating better adherence.

Duration to complete questionnaires was recorded. When a participant voiced subjective concerns or comments relating to MRQoL-LS v.1. question-stems or “troublesome” medications, they were documented by the observer. This assessment was subjective, involved only one observer and was not scrutinized using a standardised qualitative analysis.

Data were analysed using SPSS^®^ statistics software version 26.0. For normally distributed data, we calculated mean and standard deviation (SD), and median and inter-quartile range (IQR) for non-Gaussian distributed data. Relationships between variables were evaluated by Pearson’s correlation coefficient and linear regression.

The Clinical Research Ethics Committee of the Cork University Teaching Hospitals approved the study.

## Results

**Fig. 1:**
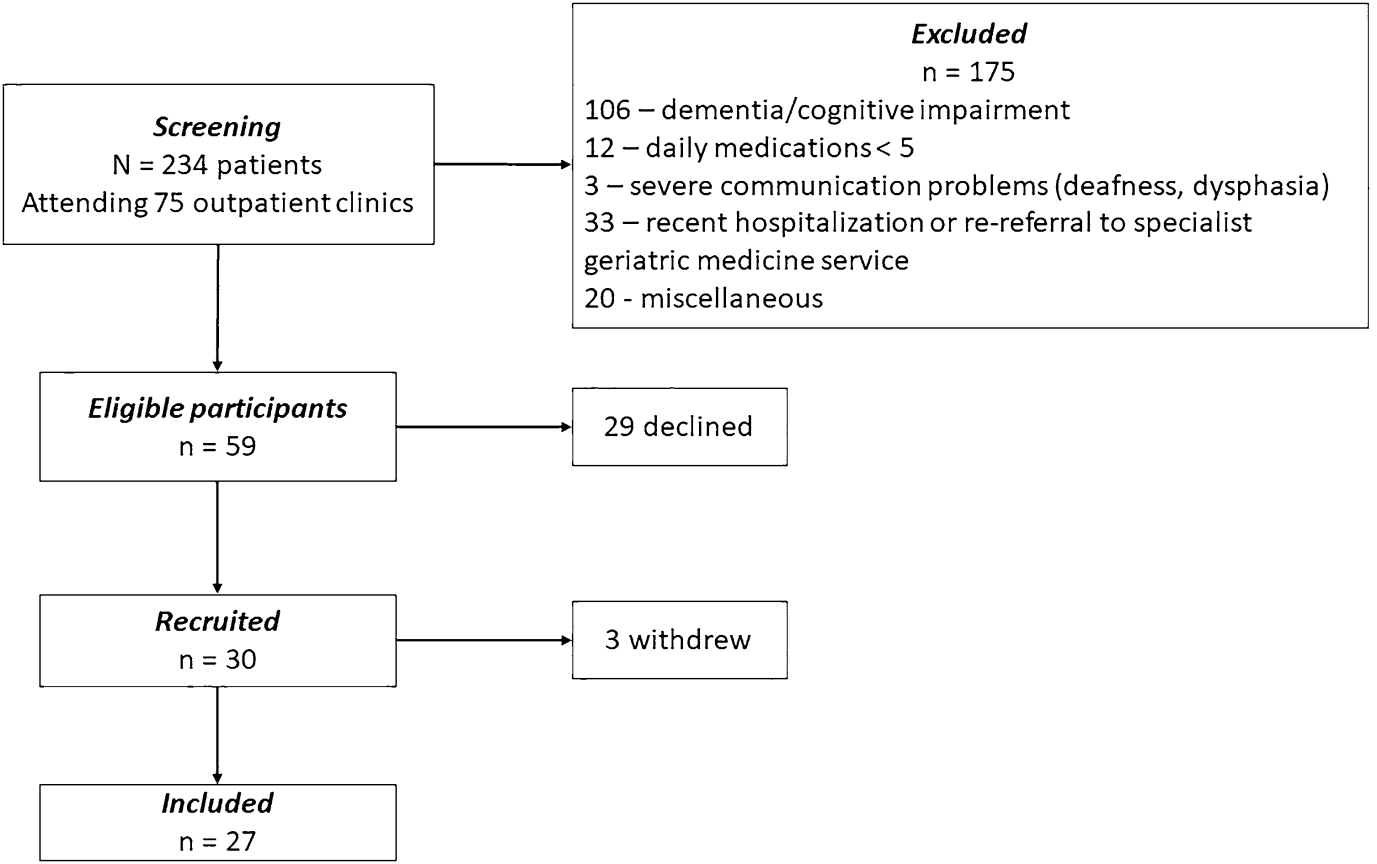
Flow diagram of patient selection

We screened 234 outpatients attending 75 consecutive geriatric ambulatory clinics, 59 patients met inclusion criteria of whom 30 patients were recruited; three patients subsequently withdrew (Fig. 1). MRQoL-LS.v.1 was largely impracticable in this cohort: only one in four patients was eligible and almost half (45%, *n* = 106) were excluded due to cognitive impairment.

Among 27 fully evaluated patients (18 female), the mean age (SD) was 80 years (5.71), their median EFS [IQR] was 4 [3–6] and their median [IQR] MMSE was 29 [28–30]. Participants had a median of 11 (IQR 9–13.5) co-morbidities and were prescribed a median of 10 (IQR 8–12.25) daily medications.

Overall, MRQoL-LS.v.1 scores were low, suggesting good medication-related QoL (median MRQoL-LS.v.1 score of 14, IQR 14–22). The median time taken to conduct an MRQoL-LS.v.1 was 2 min (range 2–5 min). Participants frequently reported difficulty differentiating some possible medication-related problems from underlying health-related problems. Of the 11 participants prescribed diuretics, three considered them as problematic medications. MRQoL and LMQ scores did not differ significantly when grouped according to presence or absence of diuretic prescriptions.

Median MARS score was 9 (IQR 6.5–10). Overall medication burden was low, the median [IQR] LMQ score was 114 [110–130] out of 300. Participants reported poorer age-adjusted HRQoL, with a mean difference SF12-PCS score (SD) of − 22.61 (11.7) and a mean difference SF12-MCS score (SD) of − 22.10 (17.43).

Higher numbers of daily medications, co-morbidities or PIMs did not correlate significantly with MRQoL-LSv.1 scores. However, poorer medication adherence (MARS score) correlated significantly with higher MRQoL-LS.v.1 scores i.e. poorer medication-related QoL (Pearson’s coefficient − 0.42, *p* = 0.04).

## Discussion

This is the first evaluation of the MRQoL-LSv.1 tool in ambulatory older adults with polypharmacy and multi-morbidity, while concurrently measuring medication burden, medication adherence and health-related QoL. Typical of this population, our cohort experienced baseline multimorbidity with polypharmacy and reported poor age-adjusted HRQoL. Despite this, overall subjectively-reported MRQoL was good. Our findings contrast with those of Tegegn et al. [10] who reported poor MRQoL in a younger Ethiopian cohort experiencing polypharmacy but with a lower comorbidity burden. Without a comparator parallel QoL assessment in that study, we can only surmise that participants genuinely had good MRQoL. Cultural, societal and psychological factors, which are not captured by MRQoL-LS.v.1., may account for these differences.

Assessment of associations between MRQoL, medication burden, HRQoL and co-morbidity burden was not possible given the small sample size. Similar to other studies the presence of PIMs was not correlated with poorer QoL [1]. Whilst frailty has previously been linked with poorer HRQoL (SF-12) [11], this was not replicated in our study. However, the majority of our cohort was not frail i.e. median (IQR) EFS score was 4 (3–6).

Our principal finding is that the utility and practicability of the MRQOL-LS.v.1. tool as an outcome measure in future research studies of multimorbid older adults is doubtful. Although quick to administer, participants reported difficulty with differentiating medication issues from more general health issues embedded in the MRQOL-LS.v.1 statements. Difficulties obtaining a larger sample size reflected practical limitations of MRQOL-LS.v.1 in real-world clinical practice, principally the need to exclude cognitively impaired patients and the absence of an MRQOL-LS.v.1 measurement by proxy. This is important, since older patients with dementia and polypharmacy are more likely to experience lower QoL [12]. Thus, MRQoL-LS.v.1. in its current form excludes a large proportion of older people who might benefit from PC interventions that could improve their QoL.

Studies using traditional HRQoL tools have failed to demonstrate improvements in QoL between interventional RCT groups despite reducing polypharmacy [13]. Future PC intervention studies should prioritise QoL reporting and preclude utilising traditional HRQoL methods, as their limited themes do not specifically assess impact of medication burden [3].

The development of an alternative more suitable MRQoL measurement tool is needed. Environment and timing of MRQoL assessment needs consideration. Community-based QoL studies predominantly show small non-significant changes with limited scope for improvement as patients already receive appropriate prescriptions, experience good overall QoL and good functional status [14]. Hospital-based PC interventions could theoretically convey substantial MRQoL improvements at a time when it matters most to the patient. Good medication adherence is associated with better QoL, therefore, future MRQoL tool design and development should prioritise patients’ medication adherence. Focussing on patients’ medication preferences and treatment goals results in improved older persons’ self-reported quality of life (EQ-VAS). Whilst subjective, more generalised QoL measurement (EQ-5D) remained unchanged, highlighting the complexity of measuring changes in wellbeing of older persons arising from particular interventions [15].

There are some limitations to our study, including; (1) its small scale which limits robust study conclusions, (2) single-centre design, (3) the lack of a direct comparator QoL tool which prevented assessment of accuracy and validity of MRQoL-LS.v.1 in the ambulatory outpatient clinical setting, and (4) variable quality of the non-standardised single observer subjective assessment of participant comments relating to MRQoL.

Geriatricians generally agree that the polypharmacy usually experienced by multi-morbid older people can have a profound effect of their QoL. Until more reliable tools for measuring QoL effects of medication in multimorbid older people are devised, MRQoL-LS.v.1 should be avoided.

## Data Availability

(Data transparency)—the included data are part of a PhD thesis awaiting submission and defence. The thesis will be available via the online CORA repository of University College Cork. Requests for further access to the data can be made via the corresponding author (EJ).

## Code availability

(Software application or custom code)—not applicable.
